# Influence of corifollitropin alfa on embryo morphokinetics and fertility treatment outcome

**DOI:** 10.3389/frph.2026.1787644

**Published:** 2026-03-25

**Authors:** Gregor Weiss, Andrea Groselj-Strele, Katharina Eberhard, Jennifer Blauensteiner, Michael Schenk

**Affiliations:** 1 Das Kinderwunsch Institut Schenk GmbH, Dobl, Austria; 2 Gottfried Schatz Research Center, Division of Cell Biology, Histology & Embryology, Medical University of Graz, Graz, Austria; 3 Core Facility Computational Bioanalytics, Medical University of Graz, Graz, Austria; 4 Department of Obstetrics and Gynecology, Medical University of Graz, Graz, Austria

**Keywords:** controlled ovarian stimulation, corifollitropin alfa, embryo morphokinetics, *in vitro* fertilization, time-lapse imaging

## Abstract

**Background:**

Corifollitropin alfa (CFA) is a long-acting recombinant follicle-stimulating hormone that reduces injection burden during controlled ovarian stimulation (COS). While its clinical efficacy has been demonstrated, its potential influence on early embryo development assessed by time-lapse morphokinetics remains insufficiently explored.

**Methods:**

This retrospective cohort study analyzed embryos derived from COS using either CFA or daily follitropin β. A total of 561 embryos from 99 CFA-stimulated patients and 3,116 embryos from 481 follitropin β-stimulated patients were included. Embryos were cultured in a time-lapse incubator, and morphokinetic parameters including pronuclear fading (tPNf) and early cleavage stages from t2 to t8 were recorded. Linear mixed-effects models were applied to account for repeated measurements per patient. Clinical outcomes including fertilization, biochemical, clinical and live birth rates were compared between groups.

**Results:**

Patients receiving CFA were older and showed lower anti-Müllerian hormone levels and higher baseline FSH concentrations, indicating a reduced ovarian reserve. No significant differences were observed between groups in morphokinetic parameters, including cleavage timings (t2–t8), cleavage synchronicity, or early developmental progression (all *p* > 0.05). The numbers of retrieved oocytes, metaphase II oocytes, and fertilization rates were comparable between stimulation protocols. Biochemical pregnancy rates were lower in the CFA group; however, clinical pregnancy and live birth rates did not differ significantly between groups.

**Conclusions:**

Controlled ovarian stimulation with CFA does not appear to affect early embryo morphokinetics as assessed by time-lapse imaging. Despite differences in baseline patient characteristics, CFA was associated with comparable embryo developmental dynamics and live birth outcomes compared with daily follitropin *β* stimulation. These findings suggest that CFA may represent an alternative COS strategy in routine clinical practice without apparent detrimental effects on early embryo development; however, they should be interpreted with caution due to the observational design.

## Graphical Abstract

**Figure:**
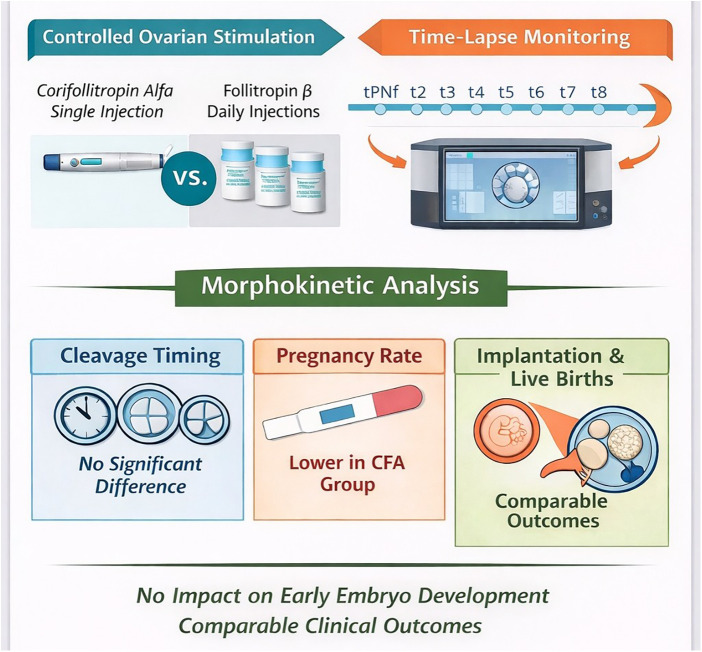
Comparison of controlled ovarian stimulation with corifollitropin alfa versus daily follitropin β. Time-lapse morphokinetic analysis showed no significant differences in early embryo cleavage timing (tPNf–t8). Although the biochemical pregnancy rate was lower in the corifollitropin alfa group, implantation, clinical pregnancy, and live birth outcomes were comparable, indicating no apparent detrimental effect on early embryo development.

## Introduction

Optimizing ovarian stimulation protocols remains a central goal in assisted reproductive technology (ART), aiming to improve clinical outcomes while reducing patient burden. One of the most significant innovations in this field is the development of long-acting recombinant follicle-stimulating hormone (FSH) analogs, such as corifollitropin alfa (CFA). CFA incorporates the carboxy-terminal peptide of the β-subunit of human chorionic gonadotropin, which prolongs its half-life to approximately 65 h while maintaining FSH receptor specificity, enabling a single injection to replace the first seven daily doses of conventional recombinant FSH (rFSH) during controlled ovarian stimulation (COS) (1–3).

Large-scale randomized clinical trials, including ENGAGE and ENSURE, have demonstrated clinical equivalence of CFA and daily rFSH in terms of oocyte yield, embryo quality, and pregnancy rates across various patient populations (1, 4). Furthermore, studies suggest that CFA is also effective in specific subpopulations such as poor responders and patients with FSH receptor polymorphisms, which may affect individual outcomes (5, 6).

Beyond the pharmacological aspects of stimulation, growing attention has been directed toward the impact of COS protocols on embryo development, particularly as assessed through time-lapse imaging technology. Time-lapse embryoscopy enables continuous, non-invasive monitoring of embryo development under stable culture conditions, allowing the capture of detailed morphokinetic parameters such as time to cleavage (t2, t3), blastocyst formation, and synchronicity of cell divisions, which have been linked to implantation potential and embryo quality (7–9).

While time-lapse culture systems offer significant laboratory advantages—improved environmental control, reduced handling, and potential for algorithmic assessment—a 2019 Cochrane review concluded that high-quality evidence on improved outcomes is still limited (10). Nevertheless, multiple studies have demonstrated associations between specific morphokinetic patterns and embryo viability, including links to chromosomal status, apoptotic gene expression in cumulus cells, and maternal age (11–15).

Recent investigations have begun to examine whether different gonadotropin regimens, such as CFA vs. daily rFSH, influence early embryo morphokinetics, potentially due to variations in the endocrine milieu during folliculogenesis. Barmat et al. and Gryshchenko et al. reported subtle but significant differences in cleavage timing (e.g., t5) and developmental speed depending on the stimulation protocol and gonadotropin dose (11, 16). These findings support the hypothesis that stimulation protocols may modulate the follicular microenvironment, oocyte competence, and subsequent embryonic behavior.

Despite advances in stimulation and embryo culture, the intersection between COS strategy and time-lapse–based embryo assessment remains incompletely understood. Only a few studies have systematically compared CFA and daily rFSH with respect to embryo morphokinetics and clinical outcomes such as implantation, ongoing pregnancy, and live birth rates (11). Addressing this gap, the present study aims to compare embryos derived from CFA and follitropin β stimulation by analyzing their morphokinetic profiles through time-lapse imaging. Furthermore, we assess clinical parameters including fertilization rate, embryo quality, pregnancy outcomes, and live birth rates to provide a comprehensive view of how gonadotropin choice may influence both early embryo development and ART success.

## Material and methods

**Figure 1:**
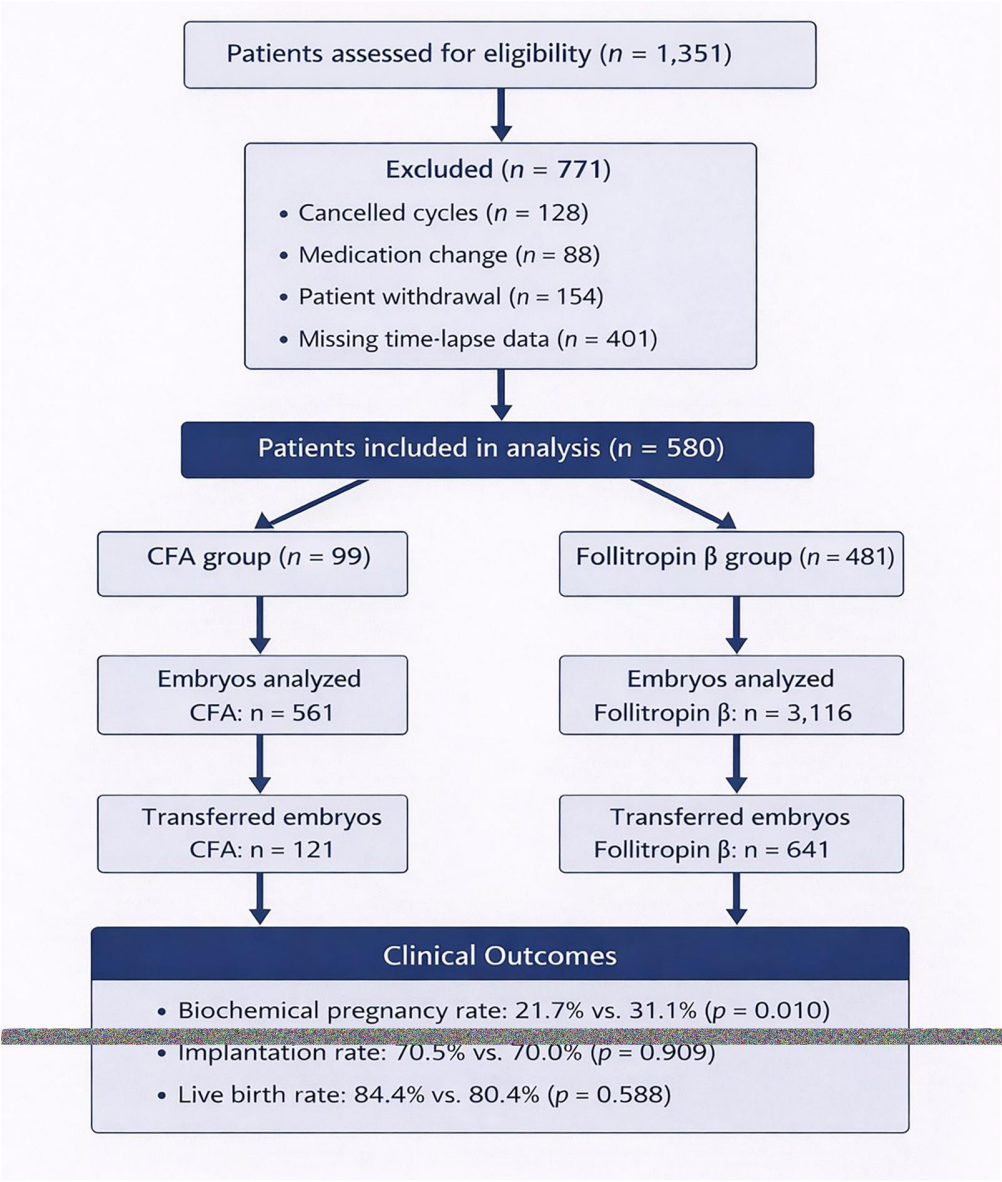
Flowchart of patient inclusion, embryo analysis, and clinical outcome assessment. Of 1,351 patients assessed for eligibility, 580 were included after exclusion of canceled cycles, medication changes, patient withdrawal, and missing time-lapse data. Ninety-nine patients received corifollitropin alfa (CFA) and 481 received daily follitropin β. A total of 561 embryos from the CFA group and 3,116 embryos from the follitropin β group were analyzed, of which 121 and 641 embryos, respectively, were transferred. The figure displays transferred embryos and not the number of embryo transfer cycles. Clinical outcomes were analyzed at the patient level.

Data were collected from the IVF institution “Das Kinderwunsch Institut Schenk GmbH” in Dobl, Austria, from 2013 to December 2018. Patients aged 18-45 years, BMI between 19 and 29.9, ovarian stimulation with CFA/follitropin β and embryos cultured in a time-lapse incubator (Embryoscope, Vitrolife AB, Göteborg, Sweden) were included in this study. Initially, a total of 1351 patients were screened for analysis. Patients were excluded because of a canceled cycle (*n* = 128), the change of medication (*n* = 86), withdrawal of the treatment/consent (*n* = 154) or missing time-lapse data (*n* = 401). From the remaining 580 patients 99 were stimulated with CFA (*n* = 561 embryos) and 481 patients were stimulated with follitropin β (*n* = 3116 embryos) (Figure 1). Informed consent was obtained from each woman with approval of the ethical committee of the Medical University of Graz (approval number 20-492ex08/09).

### Ovarian stimulation and oocyte retrieval

All patients underwent GnRH antagonist protocol COH using r-hFSH preparations CFA (Elonva®, MSD Sharp &Dohme GmbH, USA) and follitropin β (Puregon®, MSD Sharp & Dohme GmbH, USA) as previously described (17). A single injection of CFA was administered at the beginning of stimulation. After 7 days a subsequent daily stimulation with follitropin β in case of further need of stimulation was performed. In the present cohort, only 16 patients received corifollitropin alfa without additional gonadotropin supplementation. The majority required supplementary daily follitropin β after day 7, reflecting routine clinical practice. Follitropin β was administered daily for 8–10 days with dosage adaption according to age, weight, sAMH concentration, and hormonal status (3, 18). Trans-vaginal sonography was performed after 5 days of stimulation, followed by controls every second day until the day of oocyte retrieval. Ultrasonographical measurement was performed using a RIC 5-9-D 4D intravaginal probe of a GE Voluson E8 BT09 ultrasound machine (both from GE Healthcare Austria GmbH). GnRH antagonist (Cetrotide, Merck KGaA) was used to avoid premature ovulation. 5,000–10,000 IU human chorionic gonadotropin (hCG) (Pregnyl, N.V. Organon) was administered subcutaneously for triggering 36 h before oocyte retrieval (3, 18). During oocyte pick-up follicles larger than 10 mm in diameter were aspirated under sedation (Propofol, Fresenius Kabi Austria GmbH; Rapifen, Janssen-Cilag Pharma GmbH) and transvaginal ultrasound guidance (GE Healthcare Austria GmbH). Follicular fluids were examined for oocytes under steady conditions of 37 °C in an IVF workstation L24E with heating stage (K-SYSTEMS Kivex Biotec A/S, Denmark).

### Fertilization and embryo time-lapse culture

ICSI was performed on all metaphase II (MII) oocytes 4–5 h after oocyte retrieval according to the laboratory' standard operating procedure. Subsequently, oocytes were cultivated in universal culture medium (Gynemed Medizinprodukte GmbH & Co. KG, Germany) until fertilization check was performed after 16–18 h. All normal fertilized embryos with two pronuclei were cultured in a time-lapse incubator (Embryoscope). Images of the developing embryo were taken every 15 min with the built-in camera of the time-lapse incubator. Morphokinetic parameters were annotated according to Ciray nomenclature (19), including pronuclear fading (tPNf) and early cleavage stages from t2 to t8 (Table 1) and were analyzed with software developed for time-lapse image analysis (Embryoviewer® software; Vitrolife AB, Sweden). Although later developmental time points (tM, tSB/tB) were available for some embryos, these parameters contained substantial missing data in this retrospective dataset. Therefore, the primary morphokinetic analysis was restricted to cleavage-stage parameters from tPNf to t8, where data completeness allowed robust modeling. All annotations were performed by experienced embryologists following standardized protocols. Inter-observer agreement was ensured through regular internal training and consensus procedures.

**Table 1:** Morphokinetic variables and proposed definitions adapted from Ciray et al. (23).

Time	Definition of expected events
t0	Time of IVF or mid-time of micro/injection (ICSI/IMSI)
tPB2	The second polar body is completely detached from the oolemma
tPN	Fertilization status is confirmed
tPNf	Time of pronuclei disappearance; tPN1f; tPN2f
t2 to t8	Two to nine discrete cells

### Clinical outcome assessment

Biochemical pregnancy was defined as a positive serum β-hCG test 14 days after embryo transfer. Clinical pregnancy was defined as ultrasound confirmation of an intrauterine gestational sac with fetal heartbeat.

### Statistical analysis

Continuous variables were reported by means ± standard deviations (SD) or 95% confidence intervals (CI), or medians with 25-percentile and 75-percentile, whereas count data were summarized using absolute frequencies and percentages. Comparisons between groups were done for categorical data by using the chi-square test or Fisher exact test. Clinical outcomes, including biochemical pregnancy, clinical pregnancy, and live birth, were analyzed at the patient level to account for the non-independence of multiple embryos derived from the same patient. Continuous variables were examined for normality by the Kolmogorov–Smirnov test and the Shapiro–Wilk test with Lilliefors significance correction as well as by visual data inspection using Q-Q plots. Relationships between continuous variables were checked with Pearson's correlation coefficients. Linear mixed effects models were performed to deal with random effects and with unequal sample sizes for time-lapse data. The linear mixed effects models were performed as restricted maximum likelihood (REML) approach. Time in minutes to reach a specific developmental stage, measured with time-lapse technology, was the dependent variable in the model. Ovarian stimulation either with CFA or follitropin β was included as fixed between-group effect and morphokinetic parameters (from tPNf to t8) as within-group effect. Later morphokinetic time points (tM and tSB/tB) were not included in the mixed-effects models due to substantial missing data and limited sample size. Interaction effects between ovarian stimulation and morphokinetic parameters were also considered in the model, with patient ID included as a person-specific random effect to account for repeated measurements from the same patient across cycles. Baseline covariates, including age, AMH, and baseline FSH, were evaluated for inclusion in the mixed-effects models. Age and AMH were included as fixed effects where statistically appropriate, whereas baseline FSH was not retained in the final models due to multicollinearity. Given the strong intercorrelation between prognostic variables and the limited number of outcome events, further multivariable modeling of clinical endpoints was not performed. A first-order autoregressive covariance structure was used for calculation of significant differences between groups and interactions. The model selection process to define the appropriate covariance structure of the repeated effect and the random effect was based on Akaike information criterion (AIC) and Bayesian information criterion (BIC), indices of relative goodness-of-fit for the linear mixed effects model, whereas the latter criterion takes the estimation of the covariance parameters more severely into account. A two-tailed *p* value of less than *p* < 0.05 was considered as statistically significant. All statistical tests were performed using SPSS version 26.0 (SPSS Inc., Chicago, IL) and GraphPad Prism version 9 (GraphPad Software, San Diego, USA) for visualizations.

## Results

### Patients’ characteristics and clinical data

Patients stimulated with CFA were significantly older than patients stimulated with follitropin β (33.5 ± 4.1 years vs. 32.5 ± 4.6 years; *p* < 0.001). But there was no significant difference in weight [63.5 (58.0–70.0) kg vs. 62.0 (56.0–70.0) kg; *p* = 0.058] or BMI [22.5 (20.4–24.7) vs. 22.0 (20.2–24.6); *p* = 0.093]. There were no differences in progesterone [0.5 (0.3–0.8) ng/mL vs. 0.5 (0.3–0.8) ng/mL; *p* = 0.131] and estradiol [38.5 (28.2–53.6) pg/mL vs. 40.0 (30.1–50.8) pg/mL; *p* = 0.410] levels at stimulation start between the two groups. FSH concentration was higher [7.3 (5.5–8.6) mIU/mL vs. 6.7 (5.4–7.9) mIU/mL; *p* < 0.001] and AMH levels were lower [2.4 (1.5–3.7) ng/mL vs. 3.4 (1.7–5.9) ng/mL; *p* < 0.001] in patients stimulated with CFA. The ratio (76.5 vs. 75.5) of primary vs. secondary infertility did not show differences between groups (*p* = 0.627). The number of retrieved oocytes varied between 1 and 29 oocytes.

### Clinical outcome

No statistical differences were observed for quantity of retrieved, MII and fertilized oocytes. A total of 3,677 embryos were analyzed. 121 embryos (21.6%) got transferred after stimulation with CFA, compared to 641 (20.6%) in the follitropin β group. In addition, percentage of frozen embryos was similar in both groups (CFA: 101 = 18%; follitropin β: 675 = 21.7%). 40 patients treated with CFA achieved a biochemical pregnancy, defined by a positive serum β-hCG test, compared to 337 patients stimulated with follitropin β (21.7% vs. 31.1%; *p* = 0.010). However, clinical pregnancy rates and live birth rates per transfer did not differ significantly between groups (Table 2).

**Table 2:** Patients’ characteristics and clinical data for patients stimulated with *Corifollitropin alfa* or *Follitropin β*.

Parameter	*Corifollitropin alfa*	*Follitropin β*	*p*
Patients’ characteristics			
Age (years)	33.48 ± 4.06	32.50 ± 4.62	<0.001
BMI (kg/m^2^)	22.5 [20.4, 24.7]	22.0 [20.2, 24.6]	0.093
Primary/ secondary infertility (%)	76.5/23.5	75.5/24.5	0.627
Clinical data			
Progesterone (ng/mL)	0.5 [0.3, 0.8]	0.5 [0.3, 0.8]	0.131
Estradiol (pg/mL)	38.5 [28.2, 53.6]	40.0 [30.1, 50.8]	0.410
FSH (mlU/mL)	7.3 [5.5, 8.6]	6.7 [5.4, 7.9]	<0.001
AMH (ng/mL)	2.4 [1.5, 3.7]	3.4 [1.7, 5.9]	<0.001
Retrieved oocytes/ cycle	11.0 [7.0, 16.0]	10 [7.0, 14.0]	0.110
Mature oocytes/cycle	9.0 [5.0, 13.0]	9.0 [6.0, 12.0]	0.216
Fertilized oocytes/cycle	7.0 [4.0, 9.0]	6.0 [4.0, 9.0]	0.127
Embryo decision Discard/Freeze/Transfer (%)	60.4/18.0/21.6	57.8/21.7/20.6	0.148
Biochemical pregnancy rate (%)	21.7	31.1	0.010
Clinical pregnancy rate (%)	70.5	70.0	0.947
Live birth rate (%)	84.4	80.4	0.588

Values are presented as mean ± SD or median [interquartile range], as appropriate. Percentages for embryo decision refer to the proportion of embryos among all analyzed embryos (CFA *n* = 561; follitropin β *n* = 3,116).

### Morphokinetic analysis

Morphokinetic parameters were assessed from tPNf to t8 using time-lapse imaging. Developmental timing differed significantly across consecutive embryonic stages (*p* < 0.001), reflecting normal embryonic progression.

**Figure 2:**
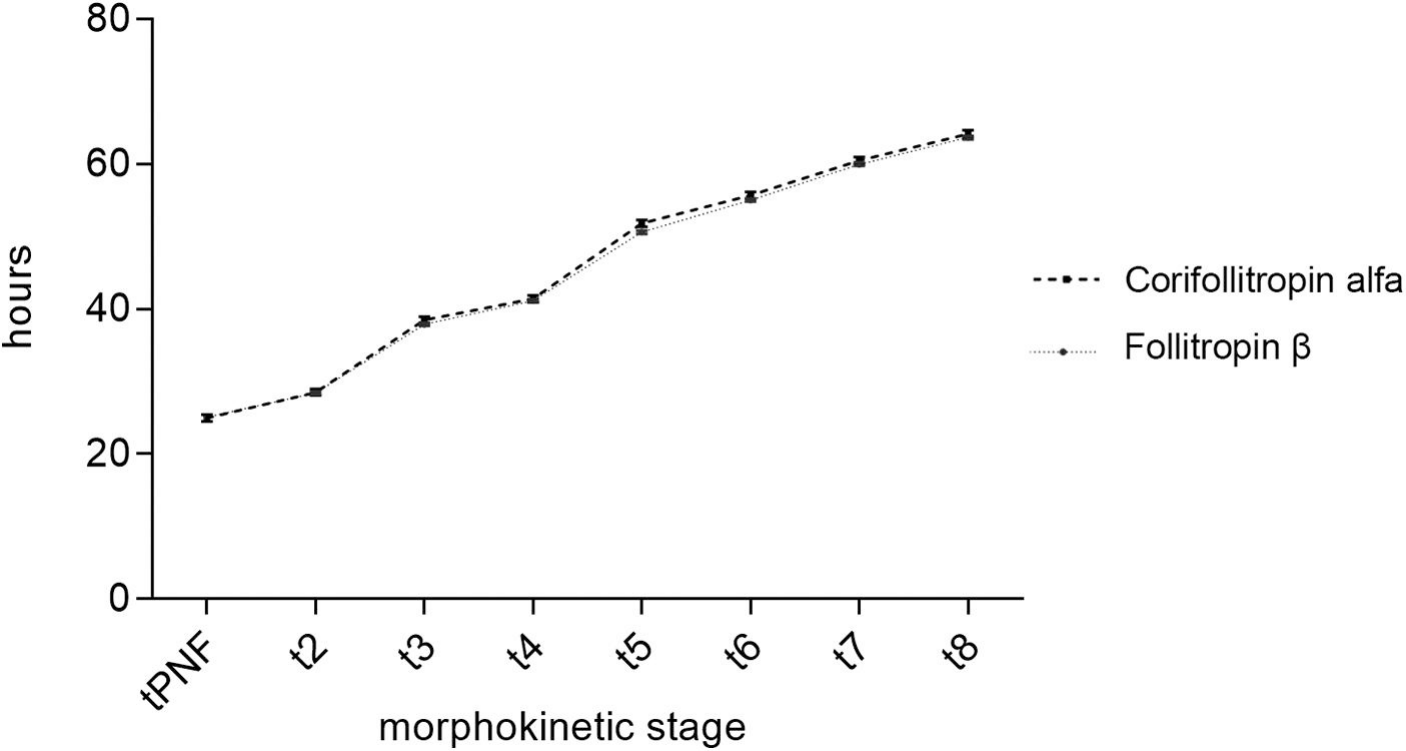
Group comparison for embryo developmental stages over time (hours) for embryos retrieved from corifollitropin alfa and follitropin β stimulated cycles. The time parameters do not significantly differ and show almost identical values.

No significant differences were observed between embryos derived from CFA and follitropin β stimulation with respect to individual morphokinetic parameters, from tPNf to t8 (main group effect: *p* = 0.284; Table 3; Figure 2).

**Table 3:** Morphokinetic parameters for patients stimulated with *Corifollitropin alfa* or *Follitropin β*.

Morphokinetic parameters	*Corifollitropin alfa n* = 561	*Follitropin β n* = 3,116
tPNf	25.07 h [24.67, 25.47]	24.95 h [ 24.06, 25.85]
t2	28.46 h [28.06, 28.86]	28.53 h [27.64, 29.43]
t3	37.89 h [37.49, 38.30]	38.49 h [37.60, 39.38]
t4	41.19 h [40.78, 41.59]	41.43 h [40.53, 42.33]
t5	50.60 h [50.20, 51.01]	51.80 h [50.89, 52.72]
t6	55.03 h [54.62, 55.44]	55.69 h [54.76, 56.62]
t7	60.00 h [59.58, 60.42]	60.47 h [59.52, 61.42]
t8	63.68 h [63.24, 64.12]	64.18 h [63.20, 65.17]

Time values are presented as mean with 95% confidence intervals (CI). tPNf (time of pronuclei disappearance); t2–t9 (two to eight discrete cells).

Infographic comparing corifollitropin alfa single injection with follitropin beta daily injections in controlled ovarian stimulation, using time-lapse monitoring and morphokinetic analysis to assess cleavage timing (no significant difference), pregnancy rate (lower in corifollitropin alfa group), and implantation or live births (comparable outcomes), concluding no impact on early embryo development and similar clinical results.

## Discussion

Using time-lapse technology, this study provides evidence that there is no difference in early cleavage-stage morphokinetics (tPNf–t8) following controlled ovarian stimulation with CFA compared to follitropin β. Additionally, we found that the number of biochemical pregnancies was higher after ovarian stimulation with follitropin β. However, the number of retrieved oocytes, MII oocytes, fertilized oocytes, as well as clinical pregnancy and live birth rates, did not differ between patients receiving daily injections of follitropin β and those treated with a single injection of CFA.

To address the global issue of infertility, IVF and ICSI were introduced, with controlled ovarian stimulation being one of the key steps in the process. Due to the relatively short half-life and rapid metabolic clearance of FSH preparations, multiple daily injections are required to stimulate follicular growth and development (20, 21). Prior to ovarian stimulation, key hormones such as progesterone and estradiol are routinely measured in clinical practice. Elevated levels of these hormones are associated with a reduced number of oocytes retrieved and lower pregnancy rates (22, 23). Additionally, AMH and FSH levels must be evaluated to avoid cycle cancellations, particularly in cases of low AMH or high FSH (24). In our study, patients treated with CFA were significantly older, had lower AMH levels, and higher FSH levels compared to those in the follitropin β group. Although the absolute age difference was relatively small and both groups were within a typical reproductive age range, the lower AMH and higher baseline FSH levels indicate a reduced ovarian reserve and less favorable baseline prognosis in the CFA group. In addition, basal progesterone and estradiol levels did not differ between the groups. Interestingly, we observed no significant differences in the number of oocytes retrieved and fertilized between the two stimulation protocols. These findings suggest that CFA induces follicular growth comparable to that of standard FSH preparations, achieving similar oocyte yields even in older patients or those with diminished ovarian reserve. A systematic review and meta-analysis previously demonstrated an increased ovarian response—and a corresponding higher risk of OHSS—in normal responders stimulated with CFA (25). Future prospective studies are required to evaluate the effects of CFA in specific responder subgroups. Given the strong intercorrelation between age, AMH, and baseline FSH in IVF populations and the limited number of outcome events, additional multivariable adjustment was carefully considered but deemed statistically unstable and potentially misleading.

In recent years, morphokinetic parameters have gained increasing interest for the evaluation and selection of embryos for transfer. Time-lapse systems enable continuous monitoring of embryo development and provide valuable tools for assessing the precise timing of early developmental events to predict successful blastocyst formation (26) based on standardized validation of morphokinetic markers (19). Successful implantation has been associated with optimal timing to the three-cell (t3) and five-cell (t5) stages (27). Deviations in morphokinetic development have been reported in embryos from women with polycystic ovary syndrome (28), aneuploid embryos (29) and patients with nicotine abuse (30). The impact of different stimulation protocols (agonist vs. antagonist) on embryo morphokinetics remains a subject of debate. In our study, we observed no significant differences in time-lapse-derived morphokinetic parameters (from tPNf to t8) between embryos obtained from cycles stimulated with either CFA or follitropin β. These findings suggest that the choice between the two stimulation agents can be guided by the individual needs of the patient, without adversely affecting early embryo development. It should be acknowledged that, although morphokinetic parameters provide valuable information on early embryo development, observed differences do not necessarily translate into improved implantation, clinical pregnancy, or live birth rates, and their predictive value may vary depending on patient characteristics and clinical context.

A significantly lower rate of biochemical pregnancies was observed in the group stimulated with CFA compared to patients stimulated with follitropin β. However, clinical pregnancy rates did not differ significantly between the groups, which is consistent with the findings of Pouwer et al., who reported no difference in clinical pregnancy rates between long-acting and daily FSH stimulation protocols (1). Furthermore, their study assessed the effectiveness of long-acting vs. daily FSH on various ART outcomes and found no differences in miscarriage rates, ectopic pregnancy rates, or multiple pregnancy rates between the two stimulation approaches. In line with these results, we also observed no difference in live birth rates between the different stimulation medications. Importantly, although a statistically significant difference was observed for biochemical pregnancy rates, this did not translate into differences in clinical pregnancy or live birth rates, indicating limited clinical relevance of this isolated finding.

Beyond treatment outcomes, the side effects and daily subcutaneous administration of FSH can represent a physical and psychological burden for patients. Feelings of depression, anxiety, isolation, and loss of control have been reported in association with fertility treatment (31). Many patients undergoing ART experience emotional distress, which has been linked to significantly lower pregnancy rates (31). Reducing the number of FSH injections—by using CFA—may offer psychological benefits for such patients.

The main strengths of this study include its clinical relevance, the large number of embryos analyzed, the standardized use of time-lapse imaging under controlled laboratory conditions, and the comparative evaluation of two widely used stimulation protocols in routine clinical practice. The incorporation of morphokinetic assessment provides continuous and objective information on early embryo development and enhances the translational value of the findings for assisted reproductive technology. In addition, linear mixed-effects models were applied to account for repeated measurements within patients, ensuring high internal consistency of the morphokinetic data.

Limitations of this study include its retrospective design, potential selection bias, unequal group sizes between stimulation protocols, and baseline differences in age and ovarian reserve parameters, which may have introduced residual confounding. Although robust statistical methods were applied, unmeasured confounders cannot be fully excluded, and age-matching was not performed. In addition, patient-reported outcomes such as psychological stress were not assessed. Furthermore, morphokinetic analyses were restricted to cleavage-stage parameters (tPNf-t8), as later developmental time points, including morula and blastocyst formation, could not be evaluated due to incomplete data and limited case numbers. Finally, most patients in the corifollitropin alfa group required additional daily FSH after day 7. Thus, the CFA group represents a combined stimulation strategy rather than a strictly isolated CFA-only protocol, which may have introduced heterogeneity in total gonadotropin exposure and endocrine milieu. Although no significant differences were observed for early morphokinetic parameters (tPNf-t8), this combined stimulation strategy should be considered when interpreting these findings, as residual confounding related to total gonadotropin exposure cannot be fully excluded. Future prospective studies with balanced cohorts and comprehensive adjustment for prognostic factors are therefore warranted.

In conclusion, our results demonstrate that morphokinetic parameters did not differ between embryos from patients stimulated with CFA compared to those stimulated with follitropin β. The number of retrieved oocytes, as well as clinical pregnancy and live birth rates, were comparable between groups. Given the observational design and baseline differences between groups, these findings should be interpreted with caution. CFA may represent a patient-friendly alternative in routine clinical practice for ovarian stimulation without apparent detrimental effects on early embryo development.

## Data Availability

The raw data supporting the conclusions of this article will be made available by the authors, without undue reservation.

## References

[1] Pouwer AW Farquhar C Kremer JAM. Long-acting FSH versus daily FSH for women undergoing assisted reproduction. Cochrane Database Syst Rev. (2015) 2015(7):CD009577. 10.1002/14651858.CD009577.pub3 26171903 PMC10415736

[2] Alvarado Franco CA Bernabeu García A Suñol Sala J Guerrero Villena J Albero Amorós S Llacer J Conventional ovarian stimulation vs. delayed single dose corifollitropin alfa ovarian stimulation in oocyte donors: a prospective randomized study. Tail trial. Front Reprod Health. (2023) 5:1239175. 10.3389/frph.2023.1239175 37965590 PMC10642283

[3] Fauser BCJM Diedrich K Devroey P, Evian Annual Reproduction Workshop Group 2007. Predictors of ovarian response: progress towards individualized treatment in ovulation induction and ovarian stimulation. Hum Reprod Update. (2008) 14(1):1–14. 10.1093/humupd/dmm034 18006561

[4] Cédrin-Durnerin I Carton I Massin N Chevalier N Dubourdieu S Bstandig B Pretreatment with luteal estradiol for programming antagonist cycles compared to no pretreatment in advanced age women stimulated with corifollitropin alfa: a non-inferiority randomized controlled trial. Hum Reprod Oxf Engl. (2024) 39(9):1979–86. 10.1093/humrep/deae167 39008826

[5] Lledó B Ortiz JA Hortal M Cascales A Morales R Guerrero J FSH receptor genotype and its influence on the results of donor ovarian stimulation using corifollitropin alfa. Reprod Biomed Online. (2022) 45(5):943–6. 10.1016/j.rbmo.2022.07.013 36075849

[6] Ferreira AF Pais AS Sousa AP Cortesão P Almeida-Santos T. Low responders may benefit from undergoing ovarian stimulation with a long GnRH agonist protocol with corifollitropin alfa followed by hMG. JBRA Assist Reprod. (2023) 27(3):414–21. 10.5935/1518-0557.20230001 37257074 PMC10712816

[7] Campbell A Fishel S Bowman N Duffy S Sedler M Hickman CFL. Modelling a risk classification of aneuploidy in human embryos using non-invasive morphokinetics. Reprod Biomed Online. (2013) 26(5):477–85. 10.1016/j.rbmo.2013.02.006 23518033

[8] Kirkegaard K Ahlström A Ingerslev HJ Hardarson T. Choosing the best embryo by time lapse versus standard morphology. Fertil Steril. (2015) 103(2):323–32. 10.1016/j.fertnstert.2014.11.003 25527231

[9] Meseguer M Herrero J Tejera A Hilligsøe KM Ramsing NB Remohí J. The use of morphokinetics as a predictor of embryo implantation. Hum Reprod. (2011) 26(10):2658–71. 10.1093/humrep/der256 21828117

[10] Armstrong S Vail A Mastenbroek S Jordan V Farquhar C. Time-lapse in the IVF-lab: how should we assess potential benefit? Hum Reprod. (2015) 30(1):38. 10.1093/humrep/deu250 25316446

[11] Maghiar L Naghi P Zaha IA Sandor M Bodog A Sachelarie L Correlation between human embryo morphokinetics observed through time-lapse incubator and life birth rate. J Pers Med. (2024) 14(10):1045. 10.3390/jpm14101045 39452552 PMC11508429

[12] VerMilyea MD Tan L Anthony JT Conaghan J Ivani K Gvakharia M Computer-automated time-lapse analysis results correlate with embryo implantation and clinical pregnancy: a blinded, multi-centre study. Reprod Biomed Online. (2014) 29(6):729–36. 10.1016/j.rbmo.2014.09.005 25444507 PMC4575500

[13] Faramarzi A Khalili MA Jahromi MG. Is there any correlation between apoptotic genes expression in cumulus cells with embryo morphokinetics? Mol Biol Rep. (2019) 46(4):3663–70. 10.1007/s11033-019-04781-z 31154602

[14] Faramarzi A Khalili MA Mangoli E. Correlations between embryo morphokinetic development and maternal age: results from an intracytoplasmic sperm injection program. Clin Exp Reprod Med. (2019) 46(3):119. 10.5653/cerm.2019.02838 31220913 PMC6736511

[15] Schenk M Groselj-Strele A Eberhard K Feldmeier E Kastelic D Cerk S Impact of polar body biopsy on embryo morphokinetics-back to the roots in preimplantation genetic testing? J Assist Reprod Genet. (2018). 10.1007/s10815-018-1207-4 29790071 PMC6086803

[16] Gryshchenko MG Pravdyuk AI Parashchyuk VY. Analysis of factors influencing morphokinetic characteristics of embryos in ART cycles. Gynecol Endocrinol. (2014) 30(sup1):6–8. 10.3109/09513590.2014.945763 25200818

[17] Schenk M Kröpfl JM Obermayer-Pietsch B Feldmeier E Weiss G. Anti-mullerian hormone concentrations in individual follicular fluids within one stimulated IVF cycle resemble blood serum values. J Assist Reprod Genet. (2017). 10.1007/s10815-017-0908-4 PMC5581778 28324271

[18] Alviggi C Humaidan P Ezcurra D. Hormonal, functional and genetic biomarkers in controlled ovarian stimulation: tools for matching patients and protocols. Reprod Biol Endocrinol RBE. (2012) 10:9. 10.1186/1477-7827-10-9 PMC3299595 22309877

[19] Ciray HN Campbell A Agerholm IE Aguilar J Chamayou S Esbert M Proposed guidelines on the nomenclature and annotation of dynamic human embryo monitoring by a time-lapse user group. Hum Reprod Oxf Engl. (2014) 29(12):2650–60. 10.1093/humrep/deu278 25344070

[20] Ben-Menahem D. Preparation, characterization and application of long-acting FSH analogs for assisted reproduction. Theriogenology. (2018) 112:11–7. 10.1016/j.theriogenology.2017.08.020 28888334

[21] Fauser BC Van Heusden AM. Manipulation of human ovarian function: physiological concepts and clinical consequences. Endocr Rev. (1997) 18(1):71–106. 10.1210/edrv.18.1.0290 9034787

[22] Listijono D Kilani S Tilia L Garrett D Chapman M. Is measurement of progesterone level prior to FSH stimulation useful in GnRH-antagonist cycles? Hum Fertil Camb Engl. (2015) 18(4):234–7. 10.3109/14647273.2015.1038658 25997693

[23] Licciardi FL Liu HC Rosenwaks Z. Day 3 estradiol serum concentrations as prognosticators of ovarian stimulation response and pregnancy outcome in patients undergoing in vitro fertilization. Fertil Steril. (1995) 64(5):991–4. 10.1016/S0015-0282(16)57916-3 7589648

[24] Revelli A Biasoni V Gennarelli G Canosa S Dalmasso P Benedetto C. IVF results in patients with very low serum AMH are significantly affected by chronological age. J Assist Reprod Genet. (2016) 33(5):603–9. 10.1007/s10815-016-0675-7 26888025 PMC4870438

[25] Mahmoud Youssef MA van Wely M Aboulfoutouh I El-Khyat W van der Veen F Al-Inany H. Is there a place for corifollitropin alfa in IVF/ICSI cycles? A systematic review and meta-analysis. Fertil Steril. (2012) 97(4):876–85. 10.1016/j.fertnstert.2012.01.092 22277766

[26] Cruz M Garrido N Herrero J Pérez-Cano I Muñoz M Meseguer M. Timing of cell division in human cleavage-stage embryos is linked with blastocyst formation and quality. Reprod Biomed Online. (2012) 25(4):371–81. 10.1016/j.rbmo.2012.06.017 22877944

[27] Basile N Vime P Florensa M Aparicio Ruiz B Garcia Velasco JA Remohi J The use of morphokinetics as a predictor of implantation: a multicentric study to define and validate an algorithm for embryo selection. Hum Reprod. (2015) 30(2):276–83. 10.1093/humrep/deu331 25527613

[28] Wissing ML Bjerge MR Olesen AIG Hoest T Mikkelsen AL. Impact of PCOS on early embryo cleavage kinetics. Reprod Biomed Online. (2014) 28(4):508–14. 10.1016/j.rbmo.2013.11.017 24581983

[29] Chawla M Fakih M Shunnar A Bayram A Hellani A Perumal V Morphokinetic analysis of cleavage stage embryos and its relationship to aneuploidy in a retrospective time-lapse imaging study. J Assist Reprod Genet. (2015) 32(1):69–75. 10.1007/s10815-014-0372-3 25395178 PMC4294870

[30] Freour T Masson D Mirallie S Jean M Bach K Dejoie T Active smoking compromises IVF outcome and affects ovarian reserve. Reprod Biomed Online. (2008) 16(1):96–102. 10.1016/S1472-6483(10)60561-5 18252054

[31] Rooney KL Domar AD. The relationship between stress and infertility. Dialogues Clin Neurosci. (2018) 20(1):41. 10.31887/DCNS.2018.20.1/klrooney 29946210 PMC6016043

